# Correction to “Metabolism‐Based Molecular Subtyping Endows Effective Ketogenic Therapy in p53‐Mutant Colon Cancer”

**DOI:** 10.1002/advs.76845

**Published:** 2026-07-31

M. Tang, F. Zhou, Q. Sun, H. Shi, et al. “Metabolism‐Based Molecular Subtyping Endows Effective Ketogenic Therapy in p53‐Mutant Colon Cancer,” *Adv* *anced* *Sci* *ence* 9 no. 29, (2022): 2201992, https://doi.org/10.1002/advs.202201992.

## Description and Source of Error

During the preparation of figures for the original publication, inappropriate cropping, resizing, and layout adjustment of Western blot images for Figure 4D,M led to visual similarities between partial bands of the two panels, which caused misunderstanding of the experimental data.

Figure 4D presents the basal expression of metabolic‐related proteins in three colon cancer cell lines (HT29, SW620, SW480). Figure 4M validates the protein knockdown efficiency of OXCT1 in SW480 cells using two independent shRNA sequences (shRNA1, shRNA3) and a negative control (NC). These two panels correspond to distinct experimental samples. In the original laboratory workflow, the two groups of samples were loaded onto the same gel and detected simultaneously on one Western blot membrane to ensure consistent experimental conditions and reliable comparison of basal protein expression. When preparing the composite figure, improper horizontal and vertical resizing and cropping of the original full Western blot image resulted in overlapping visual features between the two panels.

To address the above issues thoroughly, the authors have completed two independent new replicate experiments for all assays shown in Figure 4M–Q, including repeated Western blot verification of OXCT1 knockdown efficiency and repeated detection of ketone body utilization capacity. All raw uncropped Western blot scans, original quantification data and detailed laboratory records of the new replicates are retained for verification.

The fully corrected Figure 4, with standardized cropping, complete panel labels and data from newly repeated experiments, is provided below as it should have appeared in the original publication. All core experimental results and scientific conclusions described in the original article are fully supported and reproducible by the new replicate data.


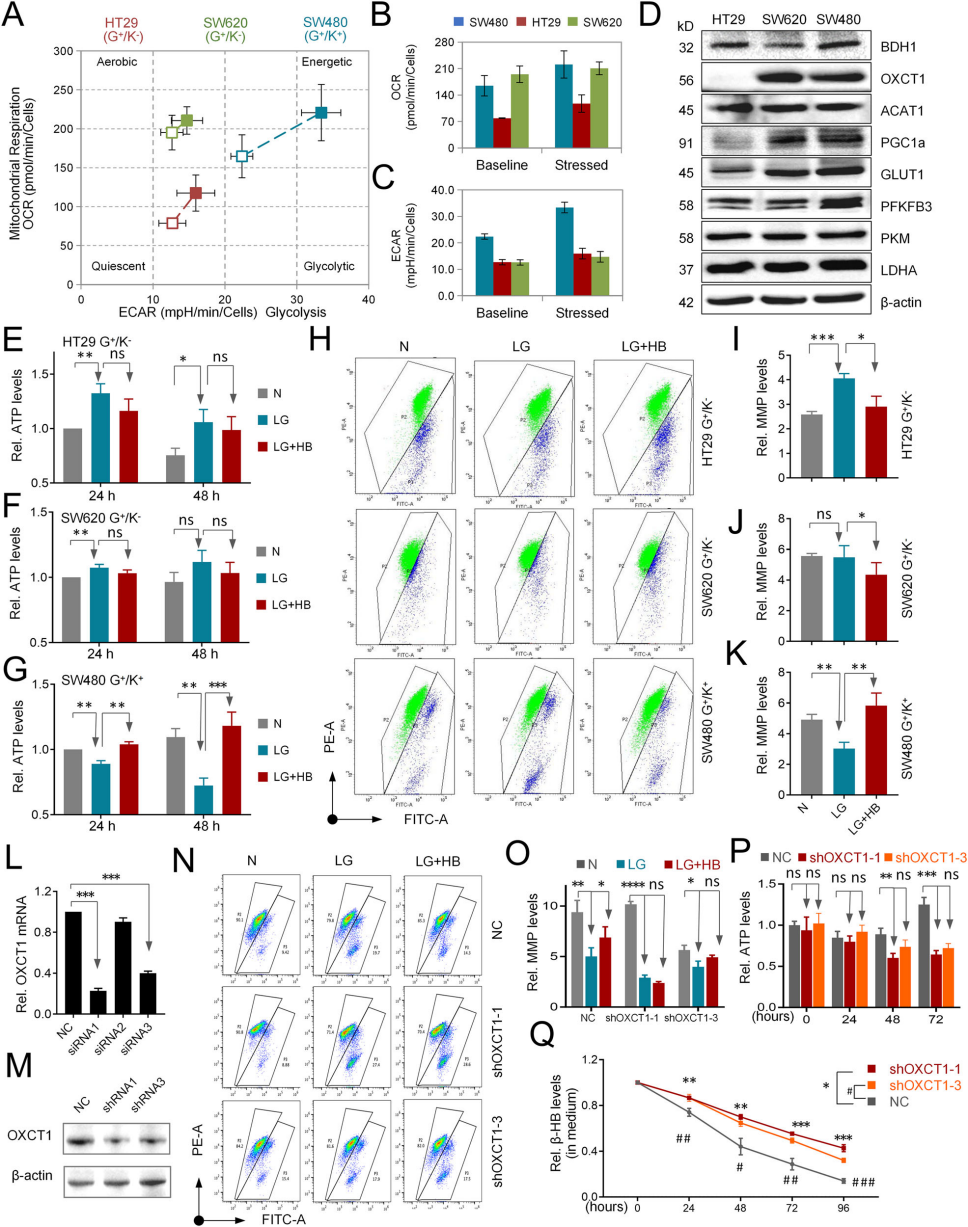


The authors apologize for these errors and for the inconvenience and confusion they may have caused to the journal, editors, reviewers, and readers.

